# The Use of Plasma-Derived Complement C1-Esterase Inhibitor Concentrate (Berinert®) in the Treatment of Angiotensin Converting Enzyme-Inhibitor Related Angioedema

**DOI:** 10.1155/2016/3930923

**Published:** 2016-03-31

**Authors:** Thorbjørn Hermanrud, Nicolaj Duus, Anette Bygum, Eva Rye Rasmussen

**Affiliations:** ^1^Department of Otorhinolaryngology and Maxillofacial Surgery, Køge Sygehus, Lykkebaekvej 1, 4600 Køge, Denmark; ^2^Department of Dermatology and Allergy Centre, Odense University Hospital, Sønder Boulevard 29, 5000 Odense C, Denmark; ^3^Department of Otorhinolaryngology, Head and Neck Surgery and Audiology, Rigshospitalet, Blegdamsvej 9, 2100 Copenhagen East, Denmark

## Abstract

Angioedema of the upper airways is a severe and potentially life-threatening condition. The incidence has been increasing in the past two decades, primarily due to pharmaceuticals influencing the generation or degradation of the vasoactive molecule bradykinin. Plasma-derived C1-esterase inhibitor concentrate is a well-established treatment option of hereditary and acquired complement C1-esterase inhibitor deficiency, which are also mediated by an increased level of bradykinin resulting in recurrent angioedema. We here present a case of severe angiotensin converting enzyme-inhibitor related angioedema (ACEi-AE) of the hypopharynx that completely resolved rapidly after the infusion of plasma-derived C1-inhibitor concentrate adding to the sparse reports in the existing literature.

## 1. Introduction

**Figure 1:**
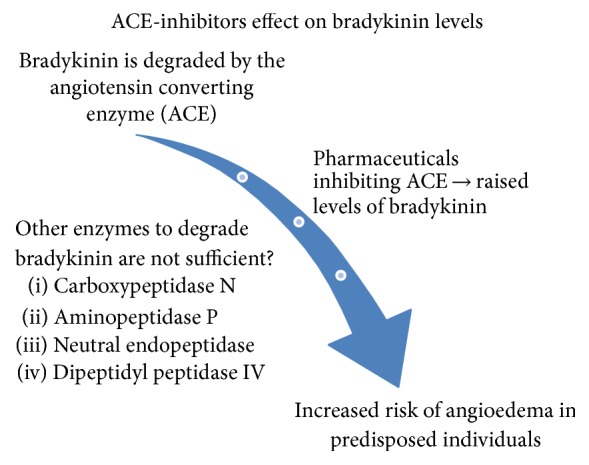
Some scientists hypothesize that patients might be predisposed to develop angioedema due to DNA mutations in the genes encoding the enzymes of the bradykinin pathway [5–10].

More than 40 million people worldwide are currently treated with angiotensin converting enzyme- (ACE-) inhibitors due to cardiovascular or renal disease [1]. The primary mechanism of angiotensin converting enzyme-inhibitor related angioedema (ACEi-AE) is accumulation of bradykinin (Figure 1). The incidence is reported to be 0.1–2.2% of exposed individuals [1, 2]. Thus, more than 400,000 cases of angioedema are to be expected worldwide yearly. ACEi-AE is a severe and potentially lethal condition due to its predilection for the subdermis and submucosa of the head and neck area, causing a serious risk of asphyxiation in these patients [3, 4]. Knowledge of the diagnosis and correct treatment options are crucial and potentially life-saving.

## 2. Case Presentation

A 56-year-old Caucasian male presented with a sensation of swelling in the throat, hoarseness, difficulty swallowing, and slight soreness in the right side of the throat. The symptoms were present when the patient awoke in the morning and progressed in a few hours, which caused him to seek medical assistance. At the local emergency department, a swollen tongue was found and the on-call otorhinolaryngologist was contacted. The patient had no known food allergies and no previous history of angioedema or urticarial eruptions. The patient did not present any signs of anaphylaxis (e.g., urticarial eruption, pruritus, hypotension, bronchospasm, or vomiting). It was unravelled that the patient 5 years ago was taking an ACE-inhibitor as an antihypertensive drug on a daily basis and thus bradykinin mediated angioedema was suspected. The patient was immediately transferred to the department of otorhinolaryngology. On admission, the symptoms were unchanged and the vital signs were normal. Lung and heart auscultation were normal. The clinical otorhinolaryngological assessment showed a moderate angioedema of the right side of the base of the tongue, the uvula, and the right palate-pharyngeal arch. Fibre-optic assessment of the pharynx showed a moderate swelling of the right side of the lingual tonsil and a severe mucosal swelling of the right side of the hypopharynx, the piriform sinus, the right aryepiglottic fold, and the right ventricular fold. The vocal folds were unaffected. Articulation was slightly impaired by the swelling. The voice was hoarse, but the respiration was unaffected. Besides a single small and sore lymph node on the right side of the neck, there were no palpable cervical lymph nodes. A bedside sonography of the neck revealed an inconspicuous lymph node with normal hilar flow in level Ib/II equivalent to the palpable lymph node. Blood samples (electrolytes, red and white blood cell count, and C-reactive protein) were normal except for a subnormal level of P-potassium 3.0 mmol/L (3.5–4.5 mmol/L). Since there were no signs of an anaphylactic reaction, tryptase was not measured. The patient did not present with symptoms and clinical findings compatible to upper airway infection (no fever, odynophagia, mucosal hyperaemia, or lymphadenitis). The patient had earlier suffered from a single deep vein thrombosis and was known to have type 2 diabetes, hypertension, hypercholesterolemia, and secondary polycythaemia due to smoking. He had no positive family history for angioedema and no prior swelling episodes. The medical history did not reveal any signs of allergic disease or malignancy. Complement analysis was not performed in the acute phase, as the biochemical analysis requires several days.

On admission, 40 milligrams of corticosteroid (Solu-Medrol®) had initially been administered intravenously. However, as the history revealed, ACEi-AE was suspected and an effect of corticosteroid could not be expected and was not awaited. Treatment with antihistamine was expected to be ineffective; thus, an antihistamine was not administrated. On suspicion of ACEi-AE, 2000 units of (18 units/kg) plasma-derived C1-inhibitor concentrate (Berinert) was administered intravenously over a course of 10 minutes. The patient rapidly reported to have decreased symptoms, but a fibre-optic reassessment was not performed until 5 hours after the infusion. A significant decrease in severity of the angioedema was observed. The patient was observed in the in-patient department for 24 hours and at discharge the angioedema had completely resolved, which was confirmed by fibre-optic reassessment. At the time of admission, the patient received an ACE-inhibitor, a calcium-antagonist, acetylsalicylic acid, a statin, and a non-beta-cell stimulating antidiabetic drug (Metformin®). The patient was thoroughly instructed never to take ACE-inhibitor again. The patient was enrolled in a large international multicenter DNA sequencing study (Prediction-ADR) in which further evaluation, including DNA testing for genetic mutations in the bradykinin pathway, is currently performed.

## 3. Discussion

As well as facial and/or oropharyngeal swelling, abdominal/intestinal pain may also occur in ACEi-AE.

Facial and/or oropharyngeal swelling may occur as part of an anaphylactic/allergic reaction which must be ruled out, since the condition can rapidly progress when not treated correctly with antiallergic medicine. In most cases, symptoms from other organs are present (e.g., urticarial eruption, hypotension, bronchospasm, and/or vomiting).

Hereditary angioedema (HAE) can be caused by excessive bradykinin formation due to complement C1-inhibitor deficiency but is also seen in patients with normal C1-inhibitor function [11]. A minor fraction of patients with normal C1-inhibitor function have mutations of factor XII and some might have estrogen dependent hereditary angioedema [11]. Seventy-five percent will have a family history of angioedema and most adults present with a history of prior angioedema attacks and/or recurrent abdominal pain episodes since childhood or early adulthood [12]. In 20–25% of patients with HAE there is no family history, as they represent* de novo* mutations [13]. The diagnosis is made on the basis of complement C1-inhibitor antigenic level and function, complement C4, and complement C1q concentration. Another differential diagnosis is acquired angioedema (AAE) most often associated with malignant diseases where increased catabolism as well as production of anti-C1-inhibitor antibodies leads to diminished levels of complement C1-inhibitor [14]. Systemic symptoms such as weight loss, fatigue, night sweats, fever, and low C1q level in association with angioedema should raise suspicion on AAE.

Other differential diagnoses to be considered are idiopathic angioedema (which rarely become life-threatening), granulomatous disease, or localised disease in the larynx, which do not have an acute onset.

The patient received acetylsalicylic acid which has angioedema listed as extremely rare adverse effect, but the ACE-inhibitor was by far the most suspicious pharmaceutical on the list.

There is no consensus regarding the treatment of ACEi-AE, but as bradykinin is the suspected offending molecule it has been proposed to use a bradykinin receptor antagonist (icatibant) which, in this case, was not available [15]. Thus, the patient was treated with plasma-derived C1-esterase inhibitor (Berinert) since there have been previous reports regarding the efficacy of this drug in severe ACE-inhibitor related angioedema [16–19]. Plasma-derived C1-esterase inhibitor concentrate is registered for treating HAE, but reports on successful outcome in patients with ACEi-AE support the use in this group of patients. Treatment with plasma-derived C1-esterase inhibitor concentrate is thought to decrease the production of bradykinin thus allowing the preexisting excess bradykinin to be degraded to inactive bradykinin metabolites [20].

Accumulation of bradykinin is responsible for angioedema episodes in HAE and AAE as well as in ACEi-AE. Drugs interfering with the pathway leading to increased levels of bradykinin are well documented in the treatment of HAE and AAE [21, 22]. However, in ACEi-AE of the head and neck only one study has described the effect of a bradykinin B2 receptor antagonist (icatibant) compared to traditional treatment with glucocorticoids and antihistamines. The median time for complete resolution in the icatibant group was 8.0 hours (3.0–16.0 hours) compared to 27.1 hours (20.3–28.0 hours) in patients treated with glucocorticoids and antihistamines [15]. Successful treatment with plasma-derived C1-inhibitor concentrate (Berinert) in patients with ACEi-AE has been described in a few case reports [16–19]. It was even found that the use of plasma-derived C1-inhibitor concentrate decreased mechanical ventilation time [23]. A study comparing the effect of a plasma-derived C1-inhibitor concentrate to glucocorticoids and antihistamines is currently being carried out (clinical trial number: NCT01843530) [24]. Other reports describe successful outcomes when treating ACEi-AE with fresh frozen plasma, but in Denmark this is not the first-choice treatment [25].

The genetic aspects of ACEi-AE are a new topic to be investigated and interesting studies have been carried out in recent years. The main challenge is to include a substantial number of patients, as the diagnosis is not always straightforward. A few mutations have, however, been identified [5–10, 26]. More studies are currently ongoing, and the patient in this case report was enrolled in a large international multicenter DNA sequencing study (Prediction-ADR), in which the genes involved in the bradykinin pathway will be assessed for disease causing polymorphisms. The patient will also be subjected to clinical follow-up (telephone interview) regarding recurrent angioedema episodes, as it is known that 11% of patients with ACEi-AE will experience relapses even when the offending medication has been discontinued [27].

Patients with ACEi-AE in the head and neck are often admitted to the emergency department where initial treatment is carried out. Knowledge of the mechanisms and treatment options in ACEi-AE is crucial since progression of the angioedema may lead to compromised airway and death [28]. Furthermore, it is very important to discontinue the medication and inform the patient of the connection, as continuation poses a severe risk of new angioedema attacks [29]. This should be followed by adequate testing necessary to exclude C1-inhibitor deficiency. As there is no test for identifying ACEi-AE, excluding C1-INH deficiency should be an essential component of the diagnostic evaluation.
